# Thermal Behavior of Poly(vinyl alcohol) in the Form of Physically Crosslinked Film

**DOI:** 10.3390/polym15081843

**Published:** 2023-04-11

**Authors:** Costas Tsioptsias, Dimitrios Fardis, Xanthi Ntampou, Ioannis Tsivintzelis, Costas Panayiotou

**Affiliations:** Department of Chemical Engineering, Aristotle University of Thessaloniki, 54124 Thessaloniki, Greece

**Keywords:** melting, decomposition, simultaneous, physical crosslinking, hydrogen bond, thermochemical transition

## Abstract

Evaluation and understanding of the thermal behavior of polymers is crucial for many applications, e.g., polymer processing at relatively high temperatures, and for evaluating polymer-polymer miscibility. In this study, the differences in the thermal behavior of poly(vinyl alcohol) (PVA) raw powder and physically crosslinked films were investigated using various methods, such as thermogravimetric analysis (TGA) and derivative TGA (DTGA), differential scanning calorimetry (DSC), Fourier transform infrared spectroscopy (FTIR) and X-ray diffraction (XRD). Various strategies were adopted, e.g., film casting from PVA solutions in H_2_O and D_2_O and heating of samples at carefully selected temperatures, in order to provide insights about the structure-properties relationship. It was found that the physically crosslinked PVA film presents an increased number of hydrogen bonds and increased thermal stability/slower decomposition rate compared to the PVA raw powder. This is also depicted in the estimated values of specific heat of thermochemical transition. The first thermochemical transition (glass transition) of PVA film, as for the raw powder, overlaps with mass loss from multiple origins. Evidence for minor decomposition that occurs along with impurities removal is presented. The overlapping of various effects (softening, decomposition, and evaporation of impurities) has led to confusion and apparent consistencies, e.g., from the XRD, it is derived that the film has decreased crystallinity, and apparently this is in agreement with the lower value of heat of fusion. However, the heat of fusion in this particular case has a questionable meaning.

## 1. Introduction

Poly(vinyl alcohol) (PVA) is a synthetic polymer which is derived from poly(vinyl acetate) [1] by hydrolysis [2]. It is an important polymer, used in the production of adhesives, emulsifiers, photographic films, food packaging materials, coatings, and others [1]. It has also been used for the production of nanocomposite materials, such as PVA matrices reinforced with montmorillonite clay [3] and graphene [4]. Its properties depend on the degree of hydrolysis and the content of the remaining acetate groups [1,5]. A high degree of hydrolysis results in an increased number of hydroxyl groups and, thus, increased hydrophilicity. Such hydrophilic behavior is of particular interest in applications that require high solubility in water, resistance to organic solvents, and strong adhesive properties to hydrophilic surfaces, such as cellulosic membranes [1]. PVA’s hydrophilicity can also be considered an advantage for improving the release of poorly water-soluble drugs [6].

In the same direction (that of hydrophilicity), PVA has been reinforced by other hydrophilic polymers, such as cellulose and chitin [7]. PVA of various hydrolysis degrees and chitosan blends have also been examined in terms of miscibility and thermal stability [5]. It was reported that, at a high degree of hydrolysis and despite the broadening of thermal transition temperature ranges, the miscibility of PVA and chitosan is poor. However, at lower degrees of hydrolysis, e.g., 88%, partial miscibility exists since the lower degree of hydrolysis results in lower crystallinity [5]. A minor broadening of the thermal transition temperatures of cellulose ester blends, consisting of cellulose acetate (CA) and cellulose acetate butyrate (CAB), was similarly shown to arise from interactions between the two polymers (enthalpy of mixing tending to zero) [8]. Such blends were characterized as heterogeneous mixtures of compatible polymers, since they could not be adequately described as fully miscible or partially miscible [8]. A similar minor broadening was also observed in the pure polymers when comparing the raw powder and films, i.e., pure polymer films exhibited a broadened thermal transition [8].

Furthermore, PVA can form gels without the use of chemical crosslinkers [9,10]. Such physically crosslinked gels are formed due to a macromolecular network that is based on hydrogen bonds. Thus, the increased capacity for hydrogen bonding has a governing influence on the gel forming ability, as well as the stability and the swelling behavior of the produced gels. The number of hydrogen bonds and, consequently, the stability and the physicochemical properties of hydrogels, can be significantly improved by freeze–thawing cycles [11,12]. PVA hydrogels have inherent self-healing properties and have been used for drug release and wound dressing applications [11].

Finally, PVA presents peculiarities in its thermal behavior. Recently, it was reported that the main thermal transition of CAB, CA and PVA had been erroneously attributed to melting; instead, this is a peculiar effect of material softening occurring simultaneously with partial decomposition [13,14]. The term *glass chemical transition* was first used to describe the inability of actual neat melting (i.e., melting without decomposition) [13], but later, the more general term *thermochemical transition* was introduced [14]. Thus, the term thermochemical transition is used for glass transition or melting that occur simultaneously with (or due to) decomposition. Apart from polymers, the same effect was recognized to occur in low molecular weight substances, such as silybin [15], quercetin [16], and gallic acid [17]. Quercetin and gallic acid were also found to depress the thermochemical transition temperature of CA membranes [18]. Other polymers, such as poly(vinyl chloride) and polypropylene-graft-maleic anhydride were reported to exhibit thermochemical transition [19]. According to literature, similar peculiarities have also been reported for other substances, e.g., for lithium potassium tartrate monohydrate [20], flavonoids (quercetin dihydrate and rutin dihydrate) [21], and potassium perchlorate [22]. The variations in the reported melting point values of succinic acid have also been attributed to the occurrence of decomposition/dehydration [23]. It is obvious that the increased capacity for hydrogen bonding is a common characteristic of the vast majority of organic substances (either polymers or low molecular weight substances) exhibiting the above-mentioned peculiarity in their thermal behavior. Recently, a study was conducted focusing on the thermal behavior of PVA raw powder with a >99% degree of hydrolysis. It was presented that the reported values for the heat of melting of PVA have a disputable physical meaning and an alternative way was proposed to quantify this heat (expressing it per mass loss and not per initial mass of the sample) [24]. The reasons of the high uncertainty of these values were also discussed [24]. At the same time, inconsistencies regarding the conclusions about the effect of heating PVA to 130 °C, which also concerned the glass transition temperature values of PVA that were reported in literature, were critically discussed [24].

The thermal behavior of PVA is of primary interest in evaluating its miscibility with other polymers [5] or for understanding its gelation mechanism [12]. However, the various reported studies did not consider the thermochemical transition. Thus, the thermal behavior of PVA could and should be reexamined under a new perspective. This can lead to a fundamental understanding of PVA’s thermal behavior and enhance the understanding of such thermal behavior observed in PVA blends and composites. Thus, the aim of this work is to study the thermal behavior of PVA in a physically crosslinked film form.

## 2. Materials and Methods

PVA raw powder (M_w_ = 89,000–98,000 g/mol and >99% hydrolyzed) was purchased from Sigma Aldrich. Deuterium oxide (D_2_O, 99.9%) was purchased from Deutero GmbH. Potassium bromide (KBr, >99.5%) was purchased from Chem-Lab. KBr was dried for 3 h at 160 °C and stored in a desiccator, while all other chemicals were used as received. Shimadzu TGA-50, Shimadzu DSC-50, Biorad FTS-175 and Brucker (model D8 advance equipped with Siemens X-ray tube, Cu, 1.54 Å) were used for thermogravimetric analysis (TGA), differential scanning calorimetry (DSC), Fourier transform infrared spectroscopy (FTIR), and X-ray diffraction (XRD) measurements, respectively. A Sartorius scale (model B 120S) with readability of 0.0001 g was also used.

PVA raw powder (1 g) was dissolved in 10 mL of water and heated for 20–30 min in the temperature range of 70–80 °C. After cooling at room temperature, the solution was cast in a petri dish and kept at 25–30 °C for 3 days to obtain the PVA film. Another film was produced using a similar procedure, however, 0.8 g of PVA raw powder was dissolved in 10 mL of heavy water (D_2_O).

PVA raw powder and the film cast from H_2_O solution were examined by TGA, DSC, XRD, and FTIR. A part of the film cast from H_2_O, which was heated up to 165 °C, and a film cast from D_2_O were also examined by FTIR. A piece of the film cast from H_2_O was weighed and then immersed in an excess of H_2_O. At various times, the sample was removed from water, carefully wiped and weighed. This procedure was repeated until weight readings stabilized. The final stable weight value was considered to be the equilibrium value of the water content of the swollen hydrogel.

DSC and TGA measurements were conducted, in aluminum and platinum pans and in the temperature range between 40–280 °C and 40–600 °C, respectively, with a heating rate of 10 K/min under nitrogen atmosphere (20 mL/min). The measurements were conducted separately and not in a simultaneous DSC-TGA instrument. The mass sample for the DSC was 1.9 mg for the PVA film and 3.1 mg for the PVA raw powder. For the TGA measurements, the initial mass of the sample was 2.6 mg for the PVA film and 6.5 mg for the raw powder.

For the FTIR measurements, the samples were mixed with KBr at mass proportion of 1/200 and processed into pellets with the aid of a hydraulic press (100 bar). FTIR measurements were performed in absorbance mode, with resolution of 2 cm^−1^ and 64 scans in the range of 400–4000 cm^−1^. XRD measurements were conducted with a step of 0.02° and for 2θ values in the range 5–50°.

Briefly, the concept of the experimental design is based on studying the sample before and after heating at carefully selected temperatures (at the end of the thermal transitions). For this reason, the film sample was studied before and after heating. Since the presence of water in PVA is expected, there will be a contribution of water in various measurements (DSC, TGA and FTIR). To provide insights for this contribution, the formation of film with D_2_O was adopted. Similar concepts from a recent work regarding the thermal behavior of gallic acid [17] proved to be useful.

## 3. Results and Discussion

**Figure 1:**
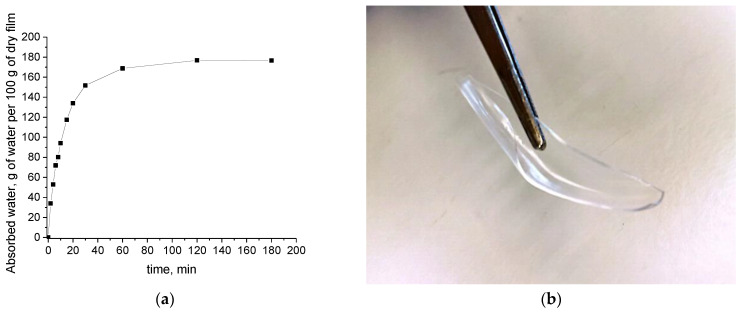
(**a**) Water absorption of the PVA film cast from H_2_O solution (expressed as g of water per g of dry film), as a function of time (the line was added only as a visual aid); (**b**) Photo of the swollen PVA film showing the retention of its initial shape.

Gelation of PVA is enhanced by the freeze–thaw process [9,11,12]. However, physical crosslinking is also achieved, to some extent, by the solution casting process and subsequent film formation. Since no crosslinker was used, the crosslinking in PVA most likely occurs in a mechanism similar to the freeze–thawing process, in which phase separation of water occurs upon cooling [11]. Similarly, water evaporation leads to a more concentrated solution, and consequently, the interactions between PVA chains are favored upon film formation. More information about the crosslinking and gelation mechanism of PVA (including schemes) can be found in a recent review [11]. The dry film (cast from H_2_O solution) exhibited the typical property of crosslinked dried gels, i.e., absorption of considerable amounts of solvent (water) and swelling while retaining its original shape. In Figure 1a, the water absorption by the dry film as a function of time is presented.

The curve in Figure 1a is presented as a simple proof that some crosslinking has occurred in the PVA film. Of course, solvent absorption and swelling may also occur in non-crosslinked polymer films but, in such a case, the original sample shape is not retained. The equilibrium value of the water content in the swollen hydrogel film is 64% wt. and, accounting for the retention of the initial shape, it is concluded that the film behaves like a gel (crosslinked structure). In Figure 1b, a photo of the PVA film at the end of the swelling experiment (containing 64% wt. water) is presented, showing the retention of its original shape.

**Figure 2:**
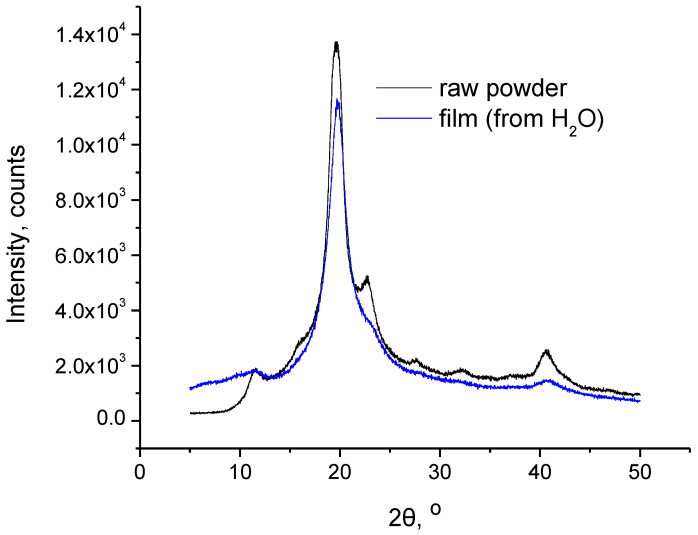
XRD patterns of PVA raw powder and film.

In Figure 2, the XRD patterns of the PVA raw powder and the film are presented. As can be seen, the same peaks exist in both samples, i.e., along with the main peak at 19.6°, a minor peak is present at 11.6 and 40.6°. At 22.7°, a shoulder peak can also be detected in the film, while the respective minor peak in the raw powder is clearly shown. These suggest that PVA exhibits the same type of crystalline structure in both raw powder and film forms. However, the decreased intensity of the peaks in the film sample as well as the practically absent peak at around 23° points to a lower degree of crystallinity. This decreased crystallinity may be related to the physical crosslinking, which could interfere with the crystallization process. However, the crosslinked film still exhibits rather high crystallinity. According to the literature [12], the initial crystallinity of PVA gels is influenced by several factors, including the concentration of PVA in the initial solution (prior to gelation). It was observed that PVA gels of increased crystallinity were formed from more concentrated PVA solutions. Thus, the rather high concentration of the solution that was used in this study may be responsible for the relatively high crystallinity of the crosslinked film.

**Figure 3:**
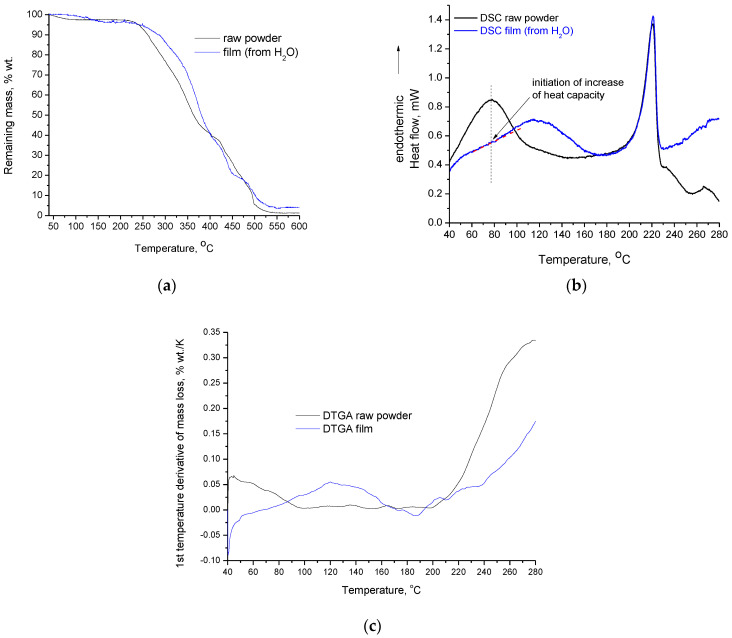
(**a**) TGA curves of PVA raw powder and PVA crosslinked film; (**b**) DSC curves of PVA raw powder and PVA crosslinked film (the pink and black dashed lines have been drawn to enable the visualization of the increase of the heat capacity); (**c**) First temperature derivative of the mass-loss curves (DTGA) of PVA raw powder and PVA crosslinked film.

In Figure 3a,b, the TGA and DSC curves of PVA raw sample and film, respectively, are presented. As shown by the TGA curves, both samples exhibit two distinct mass loss steps, one in the range of 50–150 °C and one that initiates at 200 °C. The first step is shifted to higher temperatures in the film sample. More precisely, the raw powder is already losing mass at 40 °C, and the remaining mass becomes stable around 110 °C; meanwhile the film sample starts to lose mass around 80 °C, and the remaining mass stabilizes around 160 °C. FTIR can provide insights about the origin of this mass loss and will be discussed at a later point in this section. In DSC, two thermal effects can also be detected for both samples.

The second mass loss step initiates at 200 °C for both samples. However, the mass loss of the film sample proceeds with a lower rate. This is evident both from the DTGA curves in Figure 3c and the slope of the TGA curves (Figure 3a) in the range of 200–280 °C. The film sample also exhibits higher residue at the end of the TGA measurement. The above (except the higher residue) are easier realizable on the first temperature derivative of mass-loss curves (DTGA) of PVA raw powder and film, presented in Figure 3c. In more detail, the DTGA curves in Figure 3c clearly show the two distinguished mass-loss steps, which can be identified by the existence of two main peaks (in the raw powder the first peak is not fully seen due to the rapid mass loss even at temperatures below 40 °C). Among the two samples, the differences in the temperature range of the first mass-loss step, as well the different mass-loss rate of the second step are clearly shown. Finally, the DTGA curves of Figure 3c confirm that the second mass-loss step initiates around 200 °C for both samples; however, the mass-loss rate of the raw powder is considerably higher and becomes almost doubled at 280 °C. Thus, the film sample appears to be more thermally stable than the raw powder, and along with the crosslinked structure, it is expected that other decomposition routes occur upon heating. In other words, the differences in the thermal stability and structure between the film and the raw powder are likely to lead to different decomposition paths, which in turn lead to different residues at the end of decomposition.

As shown in Figure 3b, which presents the DSC curves of both samples, there are two main endothermic effects that partially overlap with the mass-loss stages detected by the TGA/DTGA. In the raw powder, this partial overlapping is more difficult to detect. Specifically, in the DSC curve, a peak with a maximum around 75 °C is present. However, in the respective DTGA curve, the complete peak is not visible; only the half peak is shown, i.e., the maximum of the peak is located at 40 °C, which means that the peak maximum is located at the beginning of the measurement. This arises from rapid mass loss due to the evaporation of impurities. Both the DSC peak and the “half” DTGA peak in the raw powder sample end in the range 100–110 °C. This shows that the thermal effect detected by the DSC is related to the mass loss, which is detected by TGA.

Furthermore, PVA raw powder does not exhibit neat melting; instead, it shows a thermochemical transition [14,24], i.e., melting that occurs simultaneously with, or due to, some decomposition. This is also evident for the film, as shown by the DSC and DTGA curves at temperatures higher than 200 °C. Thus, the heat corresponding to the endothermic peak is also related to the heat required for the decomposition and, consequently, the values for the heat of fusion that are reported in literature are disputable. To enable the discussion, the heats of fusion were calculated through the established method (dividing the measured heat in DSC by the overall mass of the sample), but the values of heat of thermochemical transition were also calculated (dividing the heat measured in DSC by the decomposed mass, considering the overall mass of the sample examined in DSC and the percentage mass loss derived from TGA over the desired temperature range), as was recently suggested [24]. The integration of the DSC peaks was performed in the range of 175–230 °C. For the same temperature range, mass loss was obtained from the respective TGA curves. The results for the specific heat calculated by the two approaches are presented in Table 1.

Seemingly, the “heat of fusion” values are in agreement with the XRD results, i.e., the film has lower crystallinity than the raw powder. However, as was reported previously [24], the heat measured by DSC is not only related to the softening of the overall mass; it is primarily related to the decomposition of a portion of the overall mass. The values of the specific heat of thermochemical transition are comparable with the values of dissociation energy of chemical bonds, but are characterized by large uncertainty for various reasons [24]. From the values presented in Table 1, it is obvious that the film exhibits a much higher specific heat of thermochemical transition than the raw powder. This is attributed to the film’s increased thermal stability, which is most likely related to the crosslinked structure. It should be noted that the increased stability of the film was confirmed by the TGA results and precisely by the DTGA results. The Y axis in Figure 3c shows that, e.g., at 280 °C the maximum temperature rate of mass-loss is higher for the raw powder, which has a temperature rate of mass-loss equal to 0.34 % wt./K, while the film sample has a respective value of 0.17% wt./K. In the TGA curves, due to different (larger) temperature scale, the difference in the slope of the two TGA curves in the range of 200–280 °C is not easily realizable. However, as was mentioned, the difference in the mass-loss rate in the DTGA curve is clear and in accordance with the values of the specific heat of thermochemical transition. In summary, the endothermic effect in the film sample around 200 °C is a thermochemical transition with lower mass loss than that of the raw sample. The lower mass loss is related to increased thermal stability, which is related in turn to the crosslinked structure. The following paragraphs examine the endothermic effect at lower temperatures.

**Figure 4:**
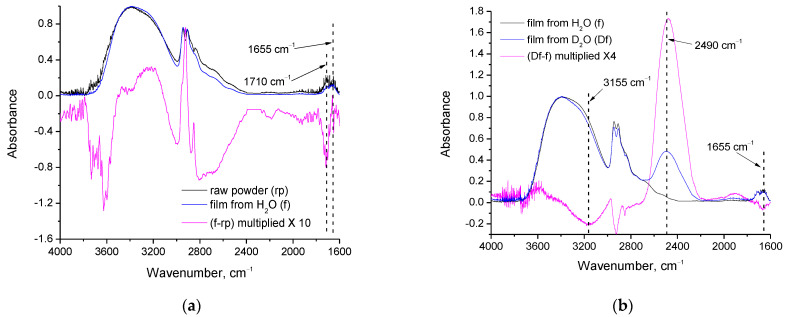

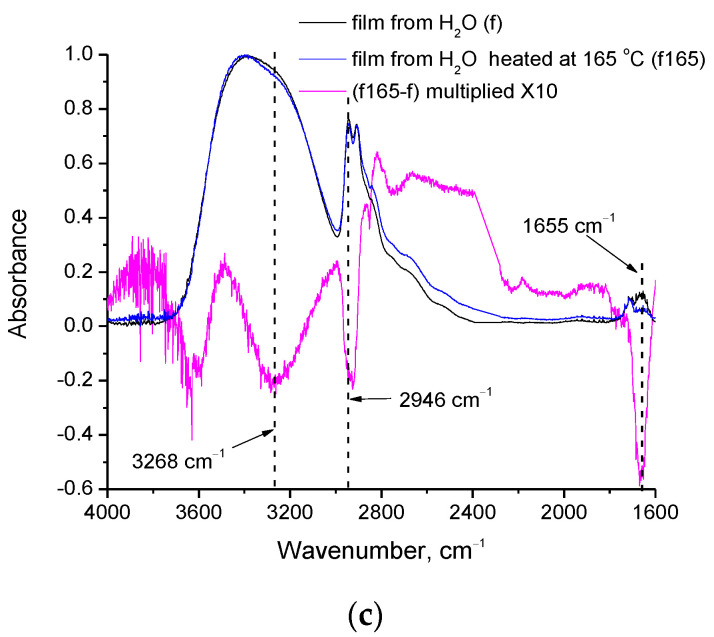
FTIR spectra and their subtracted spectrum of: (**a**) PVA raw powder and film; (**b**) film from H_2_O and film from D_2_O; (**c**) film from H_2_O before and after heating at 165 °C.

Typical values for the glass transition of PVA are in the range of 75–85 °C. As indicated by the dashed line in Figure 3b, the maximum of the peak of the raw powder (at 77 °C) coincides with the initiation of a minor increase of the heat capacity in the film. This increase of heat capacity at 77 °C is most likely caused by the glass transition. Interestingly, in both the raw powder and the film forms, the glass transition overlaps with the endothermic peaks related to mass loss. The origin of the mass loss in the raw powder was recently investigated; various contributions were reported, including evaporation of impurities such as vinyl acetate, methanol, and methyl acetate from synthesis, absorbed water, and decomposition [24]. The origin of the mass loss in the film form was investigated in this study. Along with the untreated film cast from H_2_O, the FTIR spectrum of the same film was measured after it had been heated at 165 °C. The temperature of 165 °C was chosen since the mass of the film sample becomes stable at this temperature, as shown by the TGA plots. At this temperature, the endothermic peak in the DSC graph has also ended and the curve has reached the baseline. A film cast from PVA solution in D_2_O was also examined by FTIR since this procedure can provide insights about the interaction of PVA with water. The FTIR spectra of these three samples (in pairs of two) along with their subtracted spectrum are presented in Figure 4.

As can be seen in Figure 4a, at 1655 cm^−1^ (wavenumber assigned to water bending vibration [25]), the absorption in subtracted spectrum has practically zero values, indicating that there is no important difference in the water content of the raw powder and the film. At 1710 cm^−1^, a small absorption can be detected in both the raw powder and the film. This band is characteristic of the C=O stretching [25]. There are two potential contributions, namely, C=O groups of impurities such as methyl acetate (byproduct of PVA synthesis [2]) and C=O groups of poly(vinyl acetate) [1] that were not hydrolyzed (it should be noted that the PVA used in this study has a degree of hydrolysis higher than 99% and, thus, some traces of C=O should exist). In the subtracted spectrum, a negative peak is observed at this wavenumber, which suggests decreased C=O content in the film compared to the content in the raw powder. This decrease can be attributed to methyl acetate evaporation (boiling point ~57 °C) during film formation or to further hydrolysis occurring during the dissolution of PVA in water. The remaining C=O groups in the film can be attributed to the residual acetyl groups of PVA. This interpretation agrees with and is further supported by the TGA/DSC results. As was discussed above, the first stage of mass-loss and the corresponding DSC peak for the film are shifted at higher temperatures. The raw powder loses mass from the beginning of the measurement (due to evaporation of the highly volatile methyl acetate-impurity), while in the film, there is no methyl acetate; this causes the mass loss to initiate at higher temperatures (around 80 °C). Furthermore, in the subtracted spectrum (Figure 4a), positive peaks can be observed around 2900 cm^−1^ (assigned to C-H stretching vibrations) and 3200 cm^−1^ (assigned to O-H stretching vibrations) and a negative peak around 3600 cm^−1^ (assigned to O-H stretching vibrations). It is known that the absorption bands of hydrogen-bonded OH groups appear at lower wavenumbers than the ones of loosely bound or free OH groups [25]. Thus, from the positive peak at 3200 cm^−1^ and the negative peak at 3600 cm^−1^, it can be concluded that the film presents fewer free OH groups and a higher number of hydrogen bonds. Thus, the decrease of OH groups is “inner”, and arises not from a decrease of the overall number of OH groups, but from the relative portion of free and bound OH groups. The decrease is small (the spectrum has been multiplied by 10 in order to make the positive and negative peaks visible). This small decrease in free OH, which can be attributed to the crosslinking, agrees with the small decrease in crystallinity that was observed by XRD and discussed above. This can be attributed to crosslinking that occurs during film formation. In the negative peak of 3600 cm^−1^, a contribution from removal of methanol traces may also exist. The minor positive peak at 2900 cm^−1^ (C-H stretching) suggests a denser structure in the film, which is also attributed to crosslinking.

In Figure 4b, the spectra of films cast from PVA solutions in H_2_O and D_2_O and their subtracted spectrum are presented. The presence of D_2_O residues in the respective film is confirmed from the strong absorption at 2490 cm^−1^ [25]. At 1665 cm^−1^ (water bending vibration) a small negative peak exists in the subtracted spectrum, which indicates that the H_2_O has decreased in the film cast from the D_2_O solution. However, the absorption band at this wavenumber is still present in the spectrum of the film from D_2_O, which suggests that H_2_O is not fully removed during dissolution/film casting, and therefore, that H_2_O must be strongly bound. The negative peak at 3155 cm^−1^ points out that some highly shifted (strongly bound) OH groups are missing in the film from D_2_O. The OH stretching vibration at this wavenumber cannot be attributed to water [25], and thus, this vibration is mainly assigned to PVA’s OH groups. Consequently, we are led to conclude that these highly shifted OH groups of PVA are reduced in the D_2_O film because such OH groups of the polymer were involved in hydrogen bonds with the H_2_O, which have been removed. The low wavenumber suggests that these OH groups of PVA should have been involved in multiple hydrogen bonds with water. This agrees with the previous conclusion that water must be strongly bound since is not fully removed by the film casting procedure. This also suggests that water, to some extent, may act as a crosslinker for PVA since water molecules can form up to four hydrogen bonds with four different OH groups of PVA.

**Figure 5:**
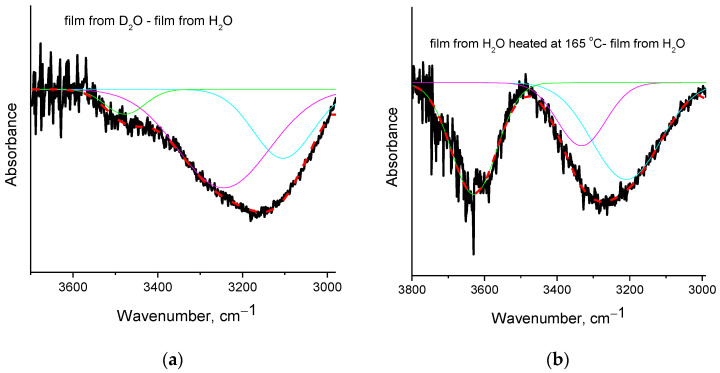
Peak fitting with Gauss peaks in the region of O-H stretching vibration: (**a**) subtracted spectrum ([Spectra of the film from D_2_O] − [Spectra of the film from H_2_O]); (**b**) subtracted spectrum ([Spectra of the film from H_2_O heated at 165 °C] − [Spectra of the film from H_2_O]). The color lines are the Gauss fitted peaks.

Regarding the differences among films from H_2_O before and after heating at 165 °C (see respective spectra in Figure 4c), a clear reduction of water content after heating is revealed from the intense negative peak at 1655 cm^−1^ (assigned to water bending vibration [25]) in the subtracted spectrum. Intense negative peaks in the C-H region (2800–3000 cm^−1^) are also observed along with various negative peaks in the O-H stretching vibration region (3100–3700 cm^−1^). In order to critically discuss these results, curve fitting (with Gauss peaks) was performed in the negative peak of the subtracted spectra ([Spectra of the film from D_2_O] − [Spectra of the film from H_2_O]) and ([Spectra of the film from H_2_O heated at 165 °C] − [Spectra of the film from H_2_O]). The fitted curves are presented in Figure 5, and the wavenumbers and percentage areas of the fitted peaks are presented in Table 2.

As can be seen in Figure 5, there are three contributions to the overall negative peak(s) in both cases. Before these three contributions are discussed, some theoretical aspects should be considered. In the PVA film, the presence of water is expected, and thus, hydrogen bonds between water and PVA’s OH groups are expected to occur. At the same time, due to the increased number of OH in PVA’s macromolecule, and since water is an impurity, the number of PVA’s OH groups is expected to be much larger than the number of water’s OH groups. Consequently, it should be expected that extensive intra- and inter-molecular hydrogen bonding will occur among the PVA’s OH groups without any involvement of water. Such OH groups should be unaffected (or slightly affected) by the removal of water and the introduction of D_2_O.

As discussed above, the negative peak in the (film from D_2_O-film from H_2_O) subtracted spectrum is centered at 3155 cm^−1^, but the fitting procedure reveals three contributions. The fitted peak at 3104 cm^−1^ represents the highly shifted OH groups of PVA that interact with water molecules (most likely involved in more than one hydrogen bonds with water). In the film from D_2_O, these OH vibrations are missing since, in that film the PVA hydroxyl groups interact with D_2_O and, thus, are shifted to much lower wavenumbers (around 2500 cm^−1^). The wavenumbers of the other two fitted peaks (at 3245 and 3478 cm^−1^) are close to the wavenumbers of the water stretching vibrations (at 3280 and 3490 cm^−1^, [25]); thus, there is a contribution from water removal in these negative peaks. However, especially in the peak with higher area (at 3245 cm^−1^) a contribution from PVA’s OH groups that are loosely bound to water, e.g., OH groups that are involved in only one hydrogen bond with water, seems to exist. More specifically, the percentage area of the peak at 3478 cm^−1^ is only 7% (Table 2), and it is shifted only by 12 cm^−1^, compared to the value of pure water at 3490 cm^−1^. Thus, this peak is mainly attributed to water. On the contrary, the peak at 3245 cm^−1^ represents the vast majority (64%) of the OH vibrations; this cannot be attributed exclusively to water since it has a larger shift compared to the value of pure water (3280 cm^−1^) and it overlaps with the main peak of pure PVA at 3340 cm^−1^ [1]. Thus, in this peak, contributions exist from both water and PVA.

A different behavior is realized in the subtracted spectrum (film from H_2_O heated at 165 °C minus film from H_2_O). Again, in the negative peak, three main partial contributions (three fitted peaks) exist. However, the wavenumbers of the fitted peaks (Table 2) are not very close to the wavenumbers of water vibrations. As discussed above, the PVA’s OH groups which interact with water should be mainly influenced upon water removal from the polymer matrix, as in the case of the film cast from D_2_O. However, from the results and analysis of the film heated at 165 °C, it is clear that water has been removed by heating (as concluded by the negative the peak at 1655 cm^−1^ shown in Figure 4c), but the OH vibrations of PVA that were affected are not only the ones due to interactions with water, but also due to PVA’s groups interacting with each other, without involvement of water. Otherwise (if just water removal occurs by heating at 165 °C), the contributions to the negative peaks of the subtracted spectra of Figure 5a,b should be quite similar. Furthermore, the intense negative peak in the C-H region suggests a decrease in organic content after heating at 165 °C. However, the film is considered free of organic impurities (as confirmed by the FTIR spectra presented in Figure 4a, the absence of early mass loss in TGA, and the related discussion), and by the film formation procedure, the only expected impurity that could shift the first stage of mass-loss to higher temperatures is water. Thus, the negative C-H peak cannot be fully attributed to removal of impurities. This negative peak, along with the negative OH peaks that cannot be attributed to PVA’s OH-water interactions, reveal the existence of a minor decomposition upon heating at 165 °C. Thus, similarly to the raw material [24], the PVA crosslinked film seems to exhibit a thermochemical transition, i.e., glass transition accompanied with, or induced by, some decomposition rather than a neat glass transition.

Finally, it is worth mentioning that the water content of the film does not seem to affect the thermal transition at high temperatures (200–250 °C), e.g., the raw sample and the film have different water content, but the transition temperature is very close in both samples. For the thermal transition at lower temperature due to the multiple overlapping thermal effects, in our opinion, no accurate determination of the softening temperature is possible by DSC; perhaps values derived from thermomechanical measurements will be more reliable.

## 4. Conclusions

The dissolution of PVA in water and casting of the subsequent solution led to the formation of a PVA film that exhibited a typical behavior of physically crosslinked structures, i.e., it can be significantly swollen in water retaining its original shape. Using FTIR, it was confirmed that, due to crosslinking, the number of bound OH groups is increased and the respective number of free OH is decreased in the film when compared to the numbers of free and bound groups in the raw powder. Crosslinking also seems to result in a small decrease of crystallinity. Furthermore, the FTIR analysis showed that the majority of PVA’s OH groups interact with each other, and only a small portion are strongly bound to water, which, to some extent, may act as crosslinker for PVA chains. Finally, due to crosslinking, the film has increased thermal stability compared to the raw powder and decomposes with a lower rate.

Organic impurities that are present in the raw powder evaporate during film formation. Consequently, in the film, the first mass-loss stage in the TGA curve and the respective DSC peak shift to higher temperatures. However, the glass transition of PVA occurs simultaneously with mass loss in both cases. The comparative FTIR study of a film heated at 165 °C and a film cast from D_2_O suggests that the origin of the mass loss cannot be exclusively attributed to evaporation of water or other impurities and should be also related to a minor decomposition. In other words, the first thermal transition of the PVA film, observed at 75–85 °C, is a thermochemical transition, i.e., glass transition accompanied with, or induced by, some minor decomposition, and not a neat glass transition. The second mass-loss stage observed in the TGA curve initiates at the same temperature with the respective DSC peak and clearly shows a second thermochemical transition exhibited by the PVA film at temperatures around 200 °C, i.e., softening/“melting” overlapping with decomposition.

## Figures and Tables

**Table 1:** Specific heat of the thermal effect in the range of 175–230 °C for PVA raw powder and film calculated by two approaches.

Sample	Mass Loss in the Range of 175–230 °C, wt.%	Specific Heat of Fusion, J/g	Specific Heat of Thermochemical Transition, J/g
Raw powder	1.1	88.3	8177
Film cast from H_2_O	0.4	71.4	17,851

**Table 2:** Wavenumber and % Area of the fitted peaks presented in Figure 5.

Fitted Peak	Film from D_2_O-Film from H_2_O		Film from H_2_O Heated at 165 °C- Film from H_2_O	
	Wavenumber, cm^−1^	% Area	Wavenumber, cm^−1^	% Area
1	3104	29	3209	45
2	3245	64	3333	21
3	3478	7	3629	34

## Data Availability

The data that support the findings of this study are available from the corresponding author upon reasonable request.
